# Novel Sulfur/Ethylenediamine-Functionalized Reduced Graphene Oxide Composite as Cathode Material for High-performance Lithium-Sulfur Batteries

**DOI:** 10.3390/nano8050303

**Published:** 2018-05-06

**Authors:** Zhuo Chen, Zhenghao Sun, Yongguang Zhang, Taizhe Tan, Yuan Tian, Zhihong Chen

**Affiliations:** 1 School of Materials Science and Engineering, Research Institute for Energy Equipment Materials, Hebei University of Technology, Tianjin 300130, China; chenzhuohebut@163.com (Z.C.); sunzhenghao666@163.com (Z.S.); 2 Synergy Innovation Institute of GDUT, Heyuan 517000, China; taizhetan@gdut.edu.cn; 3 Shenyang Institute of Automation in Guangzhou, Chinese Academy of Sciences, Guangzhou 511458, China

**Keywords:** ethylenediamine, reduced graphene oxides, lithium-sulfur batteries

## Abstract

Sulfur/ethylenediamine-functionalized reduced graphene oxide (S/EDA-RGO) nanocomposites were synthesized using a simple process. Ethylenediamine (EDA) was employed as both the reducing agent and the modification component. The morphologies, microstructures, and compositions of S/EDA-RGO composites were characterized by various detection techniques. The data indicated that EDA-RGO used as scaffolds for sulfur cathodes could enhance the electronic conductivity of the composites and strengthen the adsorbability of polysulfides. Meanwhile, the electrochemical properties of both S/EDA-RGO and S/RGO composites that were used as cathodes for lithium-sulfur (Li-S) batteries were investigated. The initial discharge capacity of S/EDA-RGO composites reached 1240 mAh g^−1^, with reversible capacity being maintained at 714 mAh g^−1^ after 100 cycles. The improvement in cycling stability of S/EDA-RGO composites was further verified at different current rates. These findings demonstrated that proper surface modification of RGO by EDA reducing agent might improve the electrochemical performances of Li–S batteries.

## 1. Introduction

Lithium-sulfur (Li-S) batteries are the most widely used renewable energy storage systems, due to their high theoretical specific capacities (around 1675 mAh g^-1^), low cost, natural abundance, and low toxicity of sulfur [1,2]. However, although these advantages benefited from high-energy rechargeable Li–S batteries, their applications as energy storage devices are still limited. First, the poor electronic conductivity of sulfur results in low utilization efficiency of sulfur cathode and poor cycle life during charge-discharge processes [3]. Second, the dissolution of polysulfides is unavoidable, leading to shuttle effect, low coulombic efficiency, poor cycle stability, and high self-discharge rate [4,5]. Besides, the large volume expansion of sulfur cathode causes fast capacity decay due to severe electrode pulverization [6,7]. 

To address these issues, various carbon materials have been proposed for the modification of sulfur cathodes to yield high-performance electrodes. These include graphene [8,9,10], carbon nanotubes [11,12,13], hollow carbon nanostructures [14,15], and mesoporous carbon [16,17]. When compared to these carbon nanomaterials, graphene is a promising two-dimensional carbon with excessive high theoretical specific surface area, superior electrical conductivity, good mechanical properties, and chemical stability. Graphene is found to be appropriate to host sulfur active materials [18,19]. Graphene-enveloped sulfur composites do not only enhance the conductivity of cathode, but also improve both the ionic and electronic transport through shorter diffusion pathways [20]. 

However, polar polysulfides often escape from nonpolar carbon frameworks. Therefore, functionalized frameworks providing significant functional groups on carbon surfaces to anchor sulfur and suppress polysulfides dissolution have been designed [21]. For instance, Manthiram et al. prepared hydroxylated graphene-sulfur nanocomposites with an improved cycling stability of Li-S batteries [22]. Furthermore, the modification of sulfur/reduced graphene oxides (S/RGO) composites with amino groups was found to be effective in binding polysulfides and yield enhanced discharge capacity and cycle performance. Among the available functional groups, we suggest that amino functionalized reduced graphene oxides-sulfur nanocomposites may prevent polysulfides dissolution in electrolytes through amino groups cross-linked to carbon host and polysulfides, while a functionalized carbon framework may effectively improve the electrical conductivity [23,24]. 

Here, a novel process was employed to synthesize sulfur/ethylenediamine-modified reduced graphene oxide (S/EDA-RGO) nanocomposites modified electrodes. Ethylenediamine (EDA) acted as both the reducing agent and the modification component. The electrochemical performances of S/EDA-RGO nanocomposites cathodes showed obvious improvements when compared to sulfur/reduced graphene oxide (S/RGO) cathodes.

## 2. Experimental 

### 2.1. Material Preparation

Graphene oxides (GO, 2 mg mL^−1^) were fabricated by the modified Hummers’ method [25,26]. Firstly, GO (15 mg) was uniformly dispersed in deionized water (30 mL) and then dehydrated ethylenediamine (EDA, ≥99.0%, 30 mL) by ultra-sonication. Secondly, the mixed solution was heated in an oil bath (80 °C) for 8 h, followed by magnetic stirring. The obtained black dispersion of EDA-RGO was freeze-dried in a vacuum oven at 60 °C for 24 h. Thirdly, the mixture was washed several times with deionized water and absolute ethyl alcohol, and then filtered off. Finally, the obtained powders of EDA-RGO were dried under vacuum at 60 °C for 8 h. The prepared 0.15 g EDA-RGO materials and 0.45 g commercial sulfur were mixed together in a mortar and were ground into powder for nearly 0.5 h. Then, the homogeneous mixture was transferred into a 10 mL stainless autoclave and was maintained at 155 °C for 12 h at argon atmosphere. After cooling down to the room temperature, the S/EDA-RGO composites were obtained. The sulfur content in the final composites was 63.5 wt %, as determined by chemical analysis (CHNS, Vario Micro Cube, Elementar, Langenselbold, Germany).

### 2.2. Characterization

The crystalline phases of the samples were conducted by X-ray diffraction (D8 Discover, Bruker, Karlsruhe, Germany) using Cu Kα radiation (λ = 0.1542 nm). The chemical transformations of the composites were detected by Fourier Transform Infrared Spectroscopy (FT-IR, Nicolet, Thermo Scientific, Waltham, MA, USA) using KBr pellet method. The Raman spectra were obtained by a Raman microscope (Thermo Scientific, Waltham, MA, USA), from 100 cm^−1^ to 3200 cm^−1^ using a laser excitation wavelength of 514 nm. X-ray photoelectron spectroscopy (XPS) measurements were analyzed with a PHI 5000 Versa Probe system (Ulvac-Phi, Kanagawa, Japan). The surface morphologies and the microstructure were examined by scanning electron microscopy (SEM, JSM-6700F, JEOL, Tokyo, Japan) and high-resolution transmission electron microscopy (HRTEM, JEM-2100F, JEOL, Tokyo, Japan).

### 2.3. Electrochemical Measurements

CR2025 coin-type cells were assembled in high purity argon-filled MBraun glove box. All of the electrochemical measurements were carried out using a Neware battery testing system (BT-2000, Arbin Instruments, College Station, TX, USA) between 1.5 V and 3 V (vs. Li/Li^+^) at room temperature. The cell consisted of S/EDA-RGO composite cathode and lithium metal anode sandwiched by microporous polypropylene membrane as the separator soaked in 75 μL electrolyte, which is a solution of 1M lithium bis (trifluoromethane) sulfonamide (LiTFSI) mixed with 1, 2-dioxolane, and dimethoxymethane (1:1 by volume). The cathode comprised of S/EDA-RGO composite, Super P conducting agent, and polyvinylidene fluoride (PVDF) as a binder (8:1:1 by weight), dissolved in N-methylpyrrolidone. The resultant slurry was uniformly spread on an aluminum foil current collector, dried at 60 °C for 12 h, and then cut into circular disks with 1 cm in diameter. The sulfur loading amount of each electrode sheet is approximately 3 mg cm^-2^.

## 3. Results and Discussion

**Figure 1:**
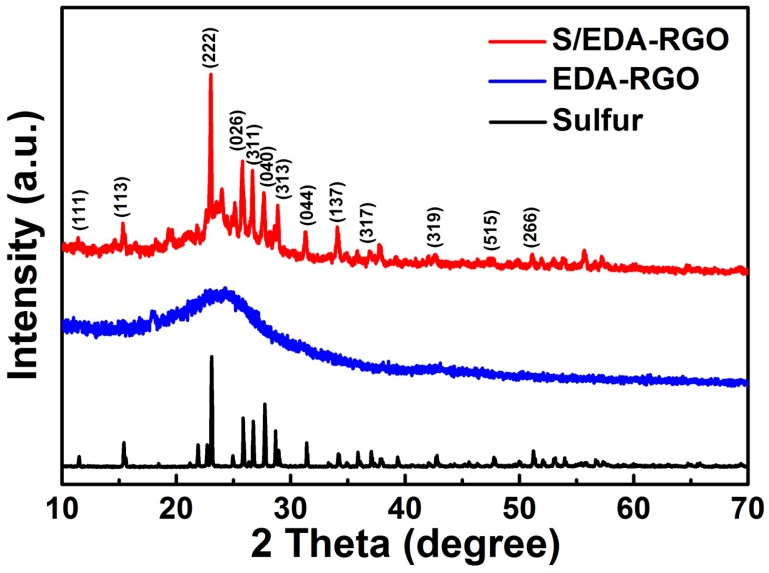
XRD patterns of Sulfur, ethylenediamine (EDA)-reduced graphene oxides (RGO), and sulfur/ethylenediamine-modified (S/EDA)-RGO composites.

To compare the microstructural properties and chemical compositions of the samples, several detection techniques, including XRD, Raman, Fourier Transform Infrared Spectroscopy (FT-IR), and X-ray photoelectron spectroscopy (XPS) were used for characterization. The XRD patterns of EDA-RGO showed broad and weak peaks that were centered at around 23.6° (Figure 1), corresponding to (002) diffraction. The absence of (001) diffraction peak centered at around 11° indicated the decreased partial reduction of graphene oxides (GO) [27]. The XRD patterns of S/EDA-RGO composite depicted a characteristic peak at 23.6°, which was assigned to RGO. The other well-resolved diffraction peaks can be indexed to the sulfur orthorhombic phase (JCPDS No. 08-0247), confirming the presence of crystallized sulfur in S/EDA-RGO composite [28,29].

**Figure 2:**
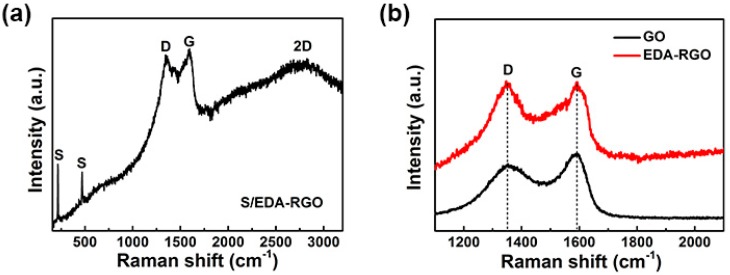
Raman spectra of S/EDA-RGO composite (**a**) and graphene oxides (GO), EDA-RGO composites (**b**).

Raman spectroscopy is a powerful non-destructive tool that is suitable for distinguishing ordered from disordered crystalline structures of carbon-based materials [30]. The Raman spectra of S/EDA-RGO composite, EDA-RGO composite, and GO are shown in Figure 2, summarizing the Raman peak positions at different ratios. The Raman spectra of S/EDA-RGO composite in Figure 2a illustrated five remarkable peaks from 100 to 3200 cm^−1^. The two sharp peaks below 600 cm^−1^ can be assigned to the S-S bond in the S/EDA-RGO composite. The three typical peaks of graphene-based materials were indexed to D band, G band, and very weak two-dimensional (2D) band at around 2700 cm^−1^ [9]. The Figure 2b suggested that all of the samples possessed two broad peaks, D band around 1353 cm^−1^ and G band around 1597 cm^−1^, corresponding to defect-induced band and crystalline graphite band, respectively [31]. In general, the ratio of D peak to G peak (*I_D_*/*I_G_*) reflects the disorder degree of carbon materials. The *I_D_*/*I_G_* value of GO was less than that of the EDA-RGO composite (0.82 vs. 1.03). This partially confirmed disordered structures after the removal of oxygen-containing functional groups and attachments of amino functional groups after the oil bath process doped into the graphene layers [32,33]. After sulfur infiltration, the *I_D_*/*I_G_* value of the S/EDA-RGO composite dropped to 0.96, demonstrating that the reaction of sulfur with amino functional groups may decrease the disorder degree of the S/EDA-RGO composite [34].

**Figure 3:**
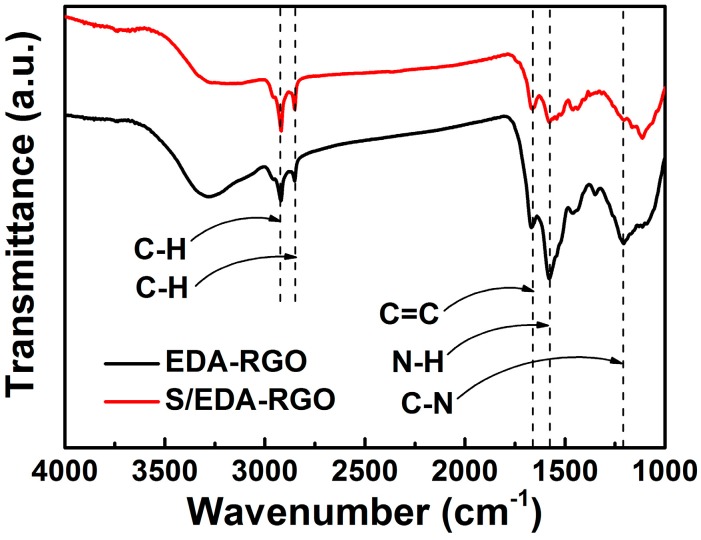
Fourier Transform Infrared Spectroscopy (FT-IR) spectra of EDA-RGO and S/EDA-RGO composites.

The FT-IR spectra of S/EDA-RGO and EDA-RGO composites are depicted in Figure 3. According to literature [35], the broad band between 3000 and 3500 cm^−1^ was assigned to O-H stretching vibration, the adsorption band at 1720 cm^−1^ was attributed to C=O stretching vibration of carboxyl or carbonyl groups, the band at 1670 cm^−1^ was associated with the overlapping absorption signals from C=C stretching vibration, and the band at 1050 cm^−1^ was linked to C-O stretching vibration [36]. For the EDA-RGO composite, the C=O and C-O peaks almost vanished, further indicating the successful partial reduction of the oxygen-containing function by ethylenediamine reaction with GO. Also, two new broad bands that were associated with N–H (1568 cm^−1^) of primary amines and aliphatic C–N (1200 cm^−1^) stretching vibrations were observed, demonstrating the successful functionalization of EDA on GO [37,38,39]. These data were consistent with the XPS analysis, confirming the successful chemical reduction and the surface modification of the EDA-RGO composite. On the other hand, intense peaks were observed at around 2852 and 2920 cm^−1^, corresponding to C-H stretching and suggesting the presence of amine functional groups on the GO surface [40].

**Figure 4:**
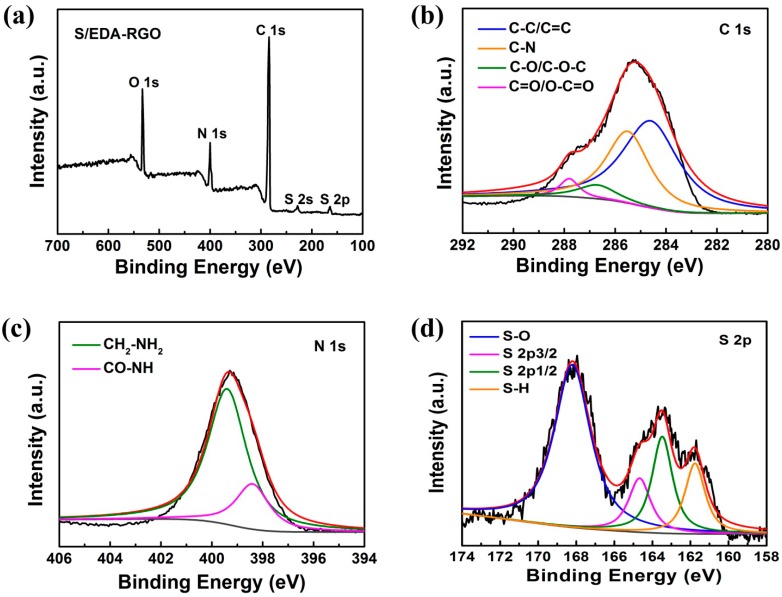
X-ray photoelectron spectroscopy (XPS) survey scan spectra (**a**), C 1s peaks (**b**), N 1s peaks (**c**), and S 2p peaks (**d**) of S/EDA-RGO composite.

The XPS spectra of S/EDA-RGO composite are depicted in Figure 4a. The characteristic peaks were observed at 533.4 eV (O 1s), 400.5 eV (N 1s), 283.9 eV (C 1s), 227.1 eV (S 2s), and 164.9 eV (S 2p). This profile confirmed the existence of nitrogen and the incorporation of sulfur in the S/EDA-RGO composite. The C 1s peak of S/EDA-RGO showed a significantly strong intensity in the XPS survey scan when compared to that of the O 1s peak. The latter clearly manifested de-oxygenation during the reduction process. The curve-fitted C 1s spectrum of S/EDA-RGO composite is presented in Figure 4b. After chemical reduction by EDA, the main peaks in C 1s spectrum of S/EDA-RGO were assigned to C-C (284.7 eV), C-O (286.7 eV), and C=O (287.8 eV), whereas the C-N peak component in amine (CH_2_-NH_2_) appeared at 285.6 eV. These features confirmed the successful reduction of GO to EDA-RGO [41]. On the other hand, the peak of C-O and C=O exhibited much weaker intensities in the S/EDA-RGO composite. This further explained the EDA functionalization on RGO that was observed with high-resolution XPS spectra based on the presence of the N1 s peak (Figure 4c). The latter could be seen from the two binding energies that were located at 398.3 eV and 399.4 eV, indicating the formation of CO–NH and CH_2_–NH_2_, respectively [42]. These data demonstrated the successful de-oxygenation by nitrogen incorporation from EDA reducing agent, which agreed well with the FT-IR data. Figure 4d displays the S2p spectra of S/EDA-RGO composite. The S 2p1/2 and S 2p3/2 peaks were located at 163.5 eV and 164.7 eV, respectively. The two other peaks that were centered at 168.2 eV and 161.7 eV could be ascribed to surface oxidation of sulfur or the interaction between sulfur and RGO [43]. The characterization by XRD, Raman, FT-IR, and XPS all suggested the successful synthesis of RGO by EDA reducing agent, along with sulfur composites.

**Figure 5:**
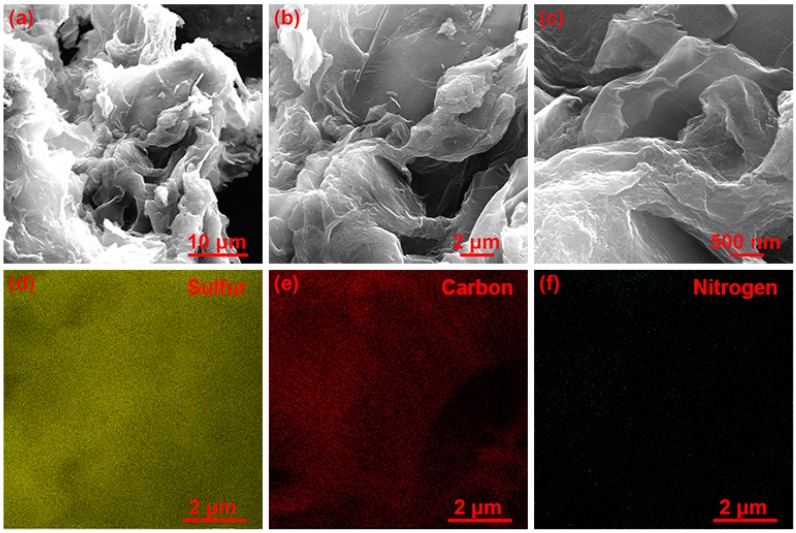
SEM images (**a**), (**b**), and (**c**) of S/EDA-RGO composites at different magnifications; EDX mapping (**d**), (**e**), and (**f**) showing distribution of sulfur, carbon, and nitrogen.

The morphology and microstructure of the S/EDA-RGO composite were carried out by SEM in Figure 5. The SEM images of the interlaced S/EDA-RGO composites showed that the sulfur was covered with the surface of EDA-RGO through van der Waals forces, which irregularly stacked together. The high-magnification SEM (Figure 5c) clearly illustrated that the S/EDA-RGO composites displayed a loose and porous nanostructure, promoting the electrolyte diffusion into the composite bulk and the electrochemical reactions. The EDX mapping of Figure 5d–f exhibited a high ratio of sulfur content and the homogeneous distribution of EDA-RGO in the S/EDA-RGO composites. It suggested that EDA-RGO could anchor sulfur and polysulfides by functional groups.

**Figure 6:**
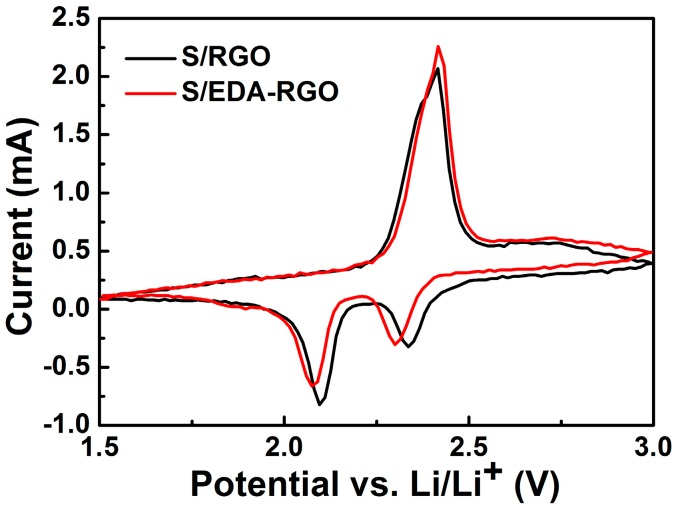
CV profiles of Li-S battery cathodes modified with EDA-RGO and S/EDA-RGO composites.

To gain a better understanding of the influence of EDA functionalized RGO in trapping polysulfides, various electrochemical measurements of Li–S battery cathodes that were based on S/RGO and S/EDA-RGO composites were carried out. Figure 6 shows the CV profiles of S/RGO and S/EDA-RGO cathodes between 1.5 V and 3 V after the first cycle at scan rate of 0.1 mV s^−1^. Distinct oxidation and reduction peaks were visible. The cathodic peaks of S/EDA-RGO that were located at around 2.3 V and 2.1 V were assigned to reduction of sulfur (S_8_) to soluble high-order lithium polysulfides (Li_2_S_n_, 8 ≥ *n* ≥ 4), and then further to insoluble lower-order lithium polysulfides Li_2_S_2_ (Li_2_S), respectively [44]. The only observed anodic peak of S/EDA-RGO at approximately 2.4 V was attributed to the conversion of Li_2_S_2_ (Li_2_S) to sulfur [45]. As can be seen from Figure 6, the redox peaks of S/EDA-RGO shifted slightly when compared to those of the S/RGO composite, indicating the relatively large polarization of the electrodes.

**Figure 7:**
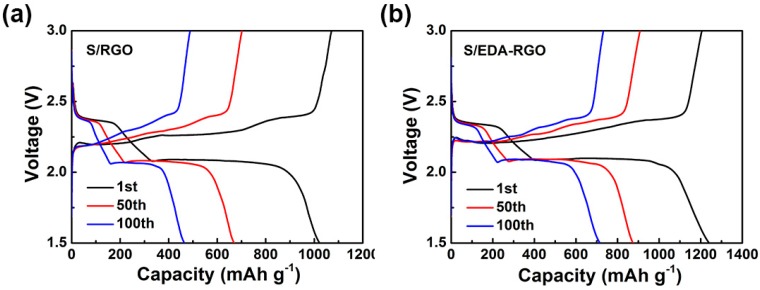
Charge/discharge curves of sulfur/reduced graphene oxides (S/RGO) (**a**) and S/EDA-RGO (**b**) composites electrodes.

Figure 7a,b exhibit the charge/discharge curves of S/RGO and S/EDA-RGO composites cathodes at 0.1 C, respectively. Obviously, the two plateaus of discharge voltage of S/EDA-RGO composites that were obtained after the 1st, 50th, and 100th cycle at 0.1 C are consistent with the two cathodic reduction peaks in CV profiles. The S/EDA-RGO voltage-capacity profiles showed slightly better electrochemical performances when compared to the S/RGO composite due to EDA functionalization, which can enhance the electronic conductivity of sulfur cathode and suppress the lithium polysulfides that were dissolved in the electrolyte. Hence, the negatively charged lithium polysulfides could be trapped tightly by the positively charged EDA [46]. Moreover, the discharge plateaus of S/EDA-RGO composites are obviously stable over 100 cycles, indicating the excellent electrochemical stability of S/EDA-RGO composites. 

**Figure 8:**
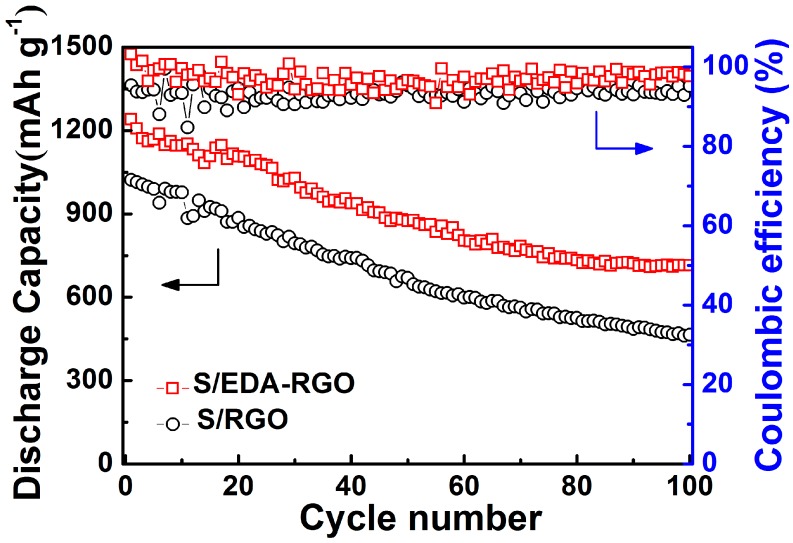
Cycling performances and corresponding coulombic efficiencies of both S/RGO and S/EDA-RGO composites electrodes at 0.1 C.

The cycling performance of S/RGO and S/EDA-RGO composites for Li–S batteries was tested between 1.5 V and 3.0 V at 0.1 C, and the results are represented in Figure 8. The discharge capacities of S/RGO and S/EDA-RGO composites were estimated to be 1021 mAh g^-1^ and 1240 mAh g^−1^ for the first cycle, respectively. The initial coulombic efficiency of S/EDA-RGO reached 100%, whereas that of S/RGO composite decreased to 95%. The S/EDA-RGO composite cathode also showed better cycling performance than unfunctionalized S/RGO. After 100 cycles, and when compared to S/RGO, the S/EDA-RGO composite revealed exceptionally stable discharge capacity of 714 mAh g^−1^ with a corresponding coulombic efficiency of 98%. Therefore, EDA functionalization effectively improved the cycling stability of the S/RGO composite cathode.

**Figure 9:**
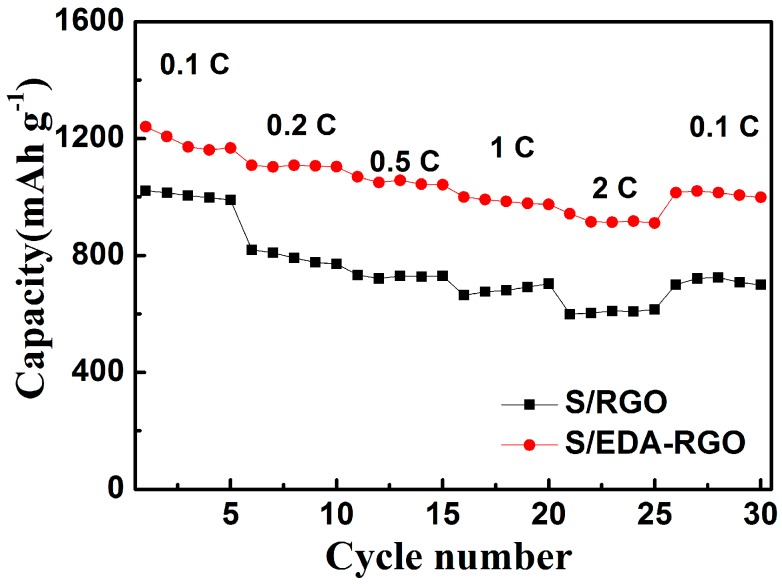
Rate performances of S/RGO and S/EDA-RGO composites electrodes.

The rate capability of S/RGO and S/EDA-RGO composites were performed at various C rates (Figure 9). The S/EDA-RGO composites delivered current densities that were reaching 1189 mAh g^−1^, 1106 mAh g^−1^, 1052 mAh g^−1^, 985 mAh g^−1^, and 932 mAh g^−1^ at 0.1 C, 0.2 C, 0.5 C, 1 C, and 2 C, respectively. After switching again to 0.1 C, the obtained discharge capacity of S/EDA-RGO composite cathode reached 1013 mAh g^−1^.

**Figure 10:**
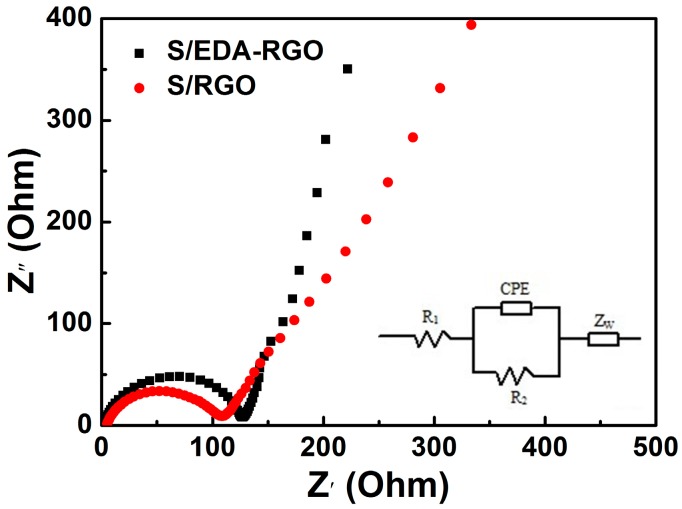
Electrochemical impedance spectroscopy (EIS) of lithium-sulfur batteries with S/RGO and S/EDA-RGO composites. The inset shows the obtained equivalent circuit.

The electrochemical impedance spectroscopy (EIS) measurements of S/RGO and S/EDA-RGO cathodes were carried out, and the data are summarized in Figure 10. Both Nyquist impedance plots exhibited semicircles at high frequency and straight line at low frequency. The equivalent circuit model is shown in the inset, including R_1_ (resistance of electrolyte and electrode), R_2_ (charge transfer resistance), CPE (constant phase capacitance), and Z_W_ (Warburg impedance) [47]. The semicircle corresponds to the charge transfer resistance, followed by a straight line of Warburg impedance for ion diffusion in the cathode. Comparison of both Nyquist impedance plots suggested that the electronic conductivity of S/EDA-RGO was higher than that of the S/RGO composite.

To confirm the enhanced electrochemical performances of EDA functionalization-modified RGO, the further comparison among S/GO, S/MWNT, S/RGO, and S/EDA-RGO cathodes of Li-S batteries were carried out and the results are compiled in Table 1. The electrochemical performances of the S/EDA-RGO composite cathode that was obtained here were better than those of the previously reported results [48,49,50]. Overall, this confirmed that EDA functionalization-modified RGO could enhance the electronic conductivity of sulfur cathode and suppress the lithium polysulfides that were dissolved in the electrolyte.

## 4. Conclusions

S/EDA-RGO composite was successfully obtained by an EDA functional-modified RGO. The EDA functionalization did not only improve the electronic conductivity of carbon framework, but also suppressed lithium polysulfides diffusion in the electrolyte. As the C rate gradually increased, the discharge capacities of S/RGO and S/EDA-RGO cathodes decreased. It will be noted that the S/EDA-RGO composite presented a steady reversible discharge capacity of 932 mAh g^−1^ at 2 C, which was higher than that of the S/RGO cathode. When the current rate returned back to 0.1 C, the capacity of S/EDA-RGO composite cathode recovered to 1013 mAh g^−1^, indicating the stable structure of S/EDA-RGO and its promise as a cathode material for Li-S batteries.

## Figures and Tables

**Table 1:** Comparison of the electrochemical performances of S/GO, S/MWNT, S/RGO, and S/EDA-RGO composite cathodes for Li-S batteries with some reported data.

Material	Initial Discharge Capacity (mAh g^−1^)	Discharge Capacity (mAh g^−1^) (after 100 Cycles)	Capacity Decay (%)	Current Density	Reference
S/GO	1053	591	43.8	0.1c	[48]
S/MWNT	1394	700	49.7	0.1c	[49]
S/RGO	1316	476	63.8	0.1c	[50]
S/EDA-RGO	1240	714	42.4	0.1c	This study
